# Experimental mixture design as a tool to optimize the growth of various *Ganoderma* species cultivated on media with different sugars

**DOI:** 10.1080/21501203.2015.1137985

**Published:** 2016-02-16

**Authors:** Yit Kheng Goh, Nurul Fadhilah Marzuki, Suet Yee Tan, Swee Sian Tan, Hun Jiat Tung, You Keng Goh, Kah Joo Goh

**Affiliations:** a Advanced Agriecological Research Sdn Bhd, No. 11 Jalan Teknologi 3/6, Taman Sains Selangor 1, Kota Damansara, Petaling Jaya, Selangor Darul Ehsan47810, Malaysia; b Applied Agricultural Resources Sdn Bhd (AAR), University of Nottingham Malaysia Campus (UNMC) Biotechnology Research Centre, Jalan Broga, Semenyih, Selangor43500, Malaysia

**Keywords:** Cluster analysis, coconut, Malaysia, mycelial growth, simple sugars, oil palm

## Abstract

The influence of different medium components (glucose, sucrose, and fructose) on the growth of different *Ganoderma* isolates and species was investigated using mixture design. Ten sugar combinations based on three simple sugars were generated with two different concentrations, namely 3.3% and 16.7%, which represented low and high sugar levels, respectively. The media were adjusted to either pH 5 or 8. *Ganoderma* isolates (two *G. boninense* from oil palm, one *Ganoderma* species from coconut palm, *G. lingzhi*, and *G. australe* from tower tree) grew faster at pH 8. *Ganoderma lingzhi* proliferated at the slowest rate compared to all other tested *Ganoderma* species in all the media studied. However, *G. boninense* isolates grew the fastest. Different *Ganoderma* species were found to have different sugar preferences. This study illustrated that the mixture design can be used to determine the optimal combinations of sugar or other nutrient/chemical components of media for fungal growth.

## Introduction

Mixture design has been adopted for use in various experiments or trials, scientific researches, and production/manufacturing lines to achieve the desired products from suitable and optimized combinations, formulations, proportions, or compositions of the inputs, materials, or components. Varieties of the products, materials, or processes are obtained or manufactured from the mixture of a few components/inputs with standardized and optimized proportions. Thus, the mixture design was widely used in different researches, studies, and industries, namely food and beverage preparations (García-García and Totosaus 2008; Kim et al. 2012), pharmaceutical trials (Cafaggi et al. 2003), development and construction sectors (Deacon et al. 1994), and manufacturing of other commercial products, such as paint, ceramic, and cement paste (Gupta 2001; Nardi et al. 2004; Ye et al. 2007).

Furthermore, mixture design was also used to optimize and produce various enzymes, antibiotics, proteins, or secondary metabolites secreted or synthesized by microbes, e.g., improving the synthesis of glycosyl hydrolase and xylanase by *Trichoderma harzianum* Rifai (Delabona et al. 2013), optimizing glutaminase production in *Bacillus* species (Sathish et al. 2008), as well as maximizing the production of other microbial compounds (Mandenius and Brundin 2008). In agricultural research, the mixture design was employed to study the growth and response of plants and crops in relation to various nutrients or by-products (Busch and Phelan 1999; Moldes et al. 2007).

Malt extract agar (MEA) amended with oil palm and coconut extracts improved mycelial growth in a few tested *Ganoderma* species, namely *G. boninense* Pat., *G. australe* (Fr.) Pat, and *G. lingzhi* Sheng H. Wu, Y. Cao & Y.C. Dai (Goh et al. 2013; Kok et al. 2013). In the same study, coconut extracts were reported to have higher sugars, in particularly, glucose, sucrose, and fructose, compared to oil palm extracts (Goh et al. 2013). Other sugars, such as galactose, lactose, and maltose, were not detected in both oil palm and coconut extracts (unpublished data). Therefore, various combinations of three different sugars, namely glucose, sucrose, and fructose, were incorporated to determine the mycelial growth rate of different *Ganoderma* species. In a few previous studies, *G. boninense, G. lucidum*, and *G. applanatum* (Pers.) Pat. grew better at around pH 5 (acidic) (Nawawi and Ho 1990; Jayasinghe et al. 2008; Jeong et al. 2009; Kapoor and Sharma 2014). In addition, oil palm extracts were reported to have pH 5 (unpublished data). *Ganoderma lucidum* was also found to proliferate in all tested pH (between pH 2 and 12). However, the growth was slower at the two extreme pHs (pH 2 and 12) (Kapoor and Sharma 2014). Among the alkaline pHs (pH 8–12) studied, pH 8 gave the highest growth (Kapoor and Sharma 2014).

In lieu of the flexible nature of the mixture design, this technique was adopted to assess the growth of different *Ganoderma* species (*G. boninense, Ganoderma* sp., *G. australe*, and *G. lingzhi*) on media with various combinations of three major simple sugars, namely glucose, sucrose, and fructose under *in vitro* conditions. The media used for this experiment were also adjusted to two separate pH: 5 (acidic) and 8 (alkaline). The objectives of this study aimed at looking for optimal sugar compositions for *Ganoderma* growth, interactions between different simple sugars and their effects on fungal growth, and sugar preference among different *Ganoderma* species (plant pathogenic species – *G. boninense, G. australe, Ganoderma* sp., and medicinal fungal species – *G. lingzhi*). Information derived from this experiment will be useful for further studies on sugar requirement by oil palm basal stem rot (BSR) pathogen, *G. boninense*, and the effect of different sugars on pathogenicity or aggressiveness of this pathogen towards oil palm.

## Materials and methods

### Fungal strains and growth conditions

Two *Ganoderma boninense* isolates (G14 and 8) from oil palms with BSR (Kok et al. 2013), one *Ganoderma* species G31 from coconut palm, one *G. australe* G30 (Goh et al. 2014), and one *G. lingzhi* G32 (fruiting body purchased from mushroom grower) were selected, maintained and grown on MEA (Difco, Becton Dickinson Diagnostics, Sparks, Maryland) supplemented with antibiotic (100 µg/L streptomycin sulphate) (Sigma-Aldrich, St Louis, Missouri) at 24°C for 7 days prior to inoculating them onto the media with various combinations of sugars.

### Mixture design and sugar compositions

Ten different combinations of sugar composition with glucose, sucrose, and fructose for both low and high sugar concentrations were generated using mixture design function in Minitab 16 (Minitab Inc., State College, PA) and the outputs or runs are outlined in Tables 1 and 2. Mixtures with low sugar media had an initial total sugar concentration of 3.3% (w/v) (Table 1), whereas the high sugar media had a total sugar concentration of 16.7% (w/v) (Table 2). pH of the mixtures with both low and high initial total sugar concentrations was adjusted to the desired pH of either 5.0 or 8.0 by adding either hydrochloric acid (0.1 M) or sodium hydroxide (0.1 M), respectively. All the media were also added with 0.25 g of MgSO_4_.7H_2_O, 0.50 g of K_2_HPO_4_, and 15 g of agar per litre of the medium. The amounts for both MgSO_4_.7H_2_O and K_2_HPO_4_ (Sigma-Aldrich, St Louis, Missouri) used were similar to the composition proposed to prepare Part A of *Ganoderma* selective medium (GSM) (Ariffin and Seman 1991). Ten-mm-diameter mycelial plugs of *Ganoderma* isolates were excised from the actively growing 7-day-old cultures using 1-cm cork-borers and placed in the centre of the tested medium. Five replicates were carried out in the study. Radial mycelial growths of all the five *Ganoderma* isolates on media with different sugar compositions and pH were measured and recorded daily for 9 days. Radial growth measurements (in mm) from the centre of the mycelial plug to the edge of each individual fungal colony were recorded. Average growth rate for individual *Ganoderma* isolates was calculated as outlined in Kok et al. (2013).

**Table 1.** Radial mycelial growth rate (mm/day) of all the selected *Ganoderma* species and isolates on the media with different sugar compositions under low sugar concentration (total of 3.3 g/100mL) at pH5 and 8.

Run number				Radial mycelial growth rate (mm/day)‡‡									
	Low sugar concentration (g/100mL)			pH 5					pH 8				
	Glucose	Sucrose	Fructose	Ga‡	Gbc	Gbp	Gbf	Gl	Ga	Gbc	Gbp	Gbf	Gl
1	3.3	0.0	0.0	0.66^B^*^(c)^†	1.69^A(b)^	2.41^A(a)^	1.50^A(b)^	0.87^ABC(c)^	1.72^CD(bc)^	1.88^A(ab)^	2.63^D(a)^	2.07^B(ab)^	0.92^A(c)^
2	1.7	0.0	1.7	0.78^B(c)^	1.32^A(b)^	2.59^A(a)^	1.57^A(b)^	0.92^AB(c)^	1.98^CD(ab)^	2.23^A(a)^	2.90^BCD(a)^	2.61^AB(a)^	0.88^A(b)^
3	0.0	0.0	3.3	1.68^A(b)^	1.53^A(b)^	2.57^A(a)^	1.81^A(b)^	0.68^BC(c)^	4.07^A(a)^	2.26^A(c)^	2.88^CD(b)^	2.49^AB(bc)^	0.83^A(d)^
4	0.0	1.7	1.7	0.71^B(c)^	1.38^A(b)^	2.64^A(a)^	1.91^A(b)^	0.68^BC(c)^	3.03^ABC(a)^	1.94^A(ab)^	3.02^BCD(a)^	2.28^B(a)^	0.81^A(b)^
5	0.0	3.3	0.0	0.81^B(d)^	1.77^A(b)^	2.81^A(a)^	1.74^A(b)^	0.61^C(d)^	2.34^CD(b)^	2.51^A(b)^	3.81^A(a)^	3.61^A(a)^	0.43^A(c)^
6	1.7	1.7	0.0	0.63^B(c)^	1.37^A(b)^	2.82^A(b)^	1.63^A(b)^	1.10^A(c)^	1.57^D(cd)^	2.27^A(bc)^	3.42^ABC(a)^	2.86^AB(ab)^	0.92^A(d)^
7	1.1	1.1	1.1	0.66^B(c)^	1.63^A(b)^	2.49^A(a)^	1.37^A(b)^	0.63^BC(c)^	3.81^AB(a)^	2.25^A(b)^	2.87^CD(ab)^	2.31^B(b)^	0.95^A(c)^
8	0.6	0.6	2.2	0.59^B(c)^	1.66^A(b)^	2.73^A(a)^	1.20^A(b)^	0.68^BC(c)^	1.87^CD(bc)^	2.53^A(ab)^	3.06^BCD(a)^	2.21^B(ab)^	0.87^A(c)^
9	0.6	2.2	0.6	0.63^B(c)^	1.70^A(b)^	2.85^A(a)^	1.56^A(b)^	0.86^ABC(c)^	2.48^BCD(b)^	2.69^A(ab)^	3.57^AB(a)^	2.54^AB(b)^	0.83^A(c)^
10	2.2	0.6	0.6	0.62^B(c)^	1.68^A(b)^	2.52^A(a)^	1.48^A(b)^	1.09^A(c)^	2.62^BCD(a)^	2.26^A(a)^	2.96^BCD(a)^	2.41^AB(a)^	0.90^A(b)^
Mean				0.78	1.57	2.64	1.58	0.81	2.55	2.28	3.11	2.54	0.83

‡‡ Each value is the mean of five replicates. ‡ *Ganoderma* isolates tested: *G. australe* G30 (Ga), two *G. boninense* isolates – G14 (Gbp) and G8 (Gbf), one *Ganoderma* species (G31), as well as *G. lingzhi* G32 (Gl). * Values in each column represent the mean radial mycelial growth rate (mm/day). Each *Ganoderma* species or isolate was analysed separately. Means within each column for the respective runs, combinations of sugar compounds, or treatments followed by the same letter (upper case letter) (superscript) are not significantly different at *P *≤ 0.05 after ANOVA–Tukey tests were performed. †Values in each horizontal row represent the mean radial mycelial growth rate (mm/day) for the respective runs or combinations of sugar compounds. Each run, combinations of sugar compounds, or treatments were analysed separately. Means within each horizontal row followed by the same letter – lower case letter, superscript, and in the bracket () – are not significantly different at *P *≤ 0.05 after ANOVA–Tukey tests were performed.

**Table 2.** Radial mycelial growth rate (mm/day) of all the selected *Ganoderma* species and isolates on the media with different sugar compositions under high sugar concentration (total of 16.7 g/100mL) at pH5 and 8.

Run number				Radial mycelial growth rate (mm/day)‡‡									
	High sugar concentration (g/100mL)			pH 5					pH 8				
	Glucose	Sucrose	Fructose	Ga‡	Gbc	Gbp	Gbf	Gl	Ga	Gbc	Gbp	Gbf	Gl
1	16.7	0.0	0.0	0.13^E^*^(c)^†	0.41^E(b)^	0.74^EF(a)^	0.30^F(b)^	0.00^B(c)^	0.12^E(c)^	0.43^E(a)^	0.52^D(a)^	0.29^D(b)^	0.05^C(c)^
2	8.3	0.0	8.3	0.47^D(a)^	0.45^DE(a)^	0.58^F(a)^	0.46^EF(a)^	0.05^B(b)^	0.47^D(bc)^	0.56^DE(ab)^	0.70^D(a)^	0.46^CD(bc)^	0.32^ABC(c)^
3	0.0	0.0	16.7	0.69^C(b)^	0.49^DE(c)^	0.97^DEF(a)^	0.92^CD(a)^	0.02^B(d)^	0.78^C(bc)^	0.57^DE(c)^	1.49^C(a)^	0.97^BC(b)^	0.15^BC(d)^
4	0.0	8.3	8.3	1.11^B(ab)^	0.82^BC(b)^	1.45^BC(a)^	1.27^BC(a)^	0.17^AB(c)^	1.21^B(b)^	1.03^B(b)^	2.32^B(a)^	1.29^B(b)^	0.35^ABC(c)^
5	0.0	16.7	0.0	1.47^A(bc)^	1.30^A(c)^	2.75^A(a)^	1.72^A(b)^	0.28^A(d)^	1.41^A(d)^	1.90^A(c)^	4.02^A(a)^	2.95^A(b)^	0.51^AB(e)^
6	8.3	8.3	0.0	0.57^CD(b)^	0.93^B(a)^	1.11^CDE(a)^	0.88^D(ab)^	0.16^AB(c)^	0.77^C/(bc)^	0.92^BC(b)^	1.52^C(a)^	0.52^CD(cd)^	0.32^ABC(d)^
7	5.5	5.5	5.5	0.72^C(b)^	0.69^BCD(b)^	1.31^CD(a)^	0.69^DE(b)^	0.17^AB(c)^	0.77^C(b)^	0.82^BCD(b)^	1.53^C(a)^	0.54^CD(bc)^	0.43^ABC(c)^
8	2.8	2.8	11.1	0.60^CD(b)^	0.62^CDE(b)^	1.01^CDEF(a)^	0.78^DE(ab)^	0.19^AB(c)^	0.89^C(b)^	0.82^BCD(b)^	1.42^C(a)^	0.66^CD(bc)^	0.41^ABC(c)^
9	2.8	11.1	2.8	1.03^B(b)^	1.19^A(b)^	1.87^B(a)^	1.36^AB(b)^	0.19^AB(c)^	1.41^A(b)^	1.07^B(bc)^	2.49^B(a)^	1.22^B(bc)^	0.66^A(c)^
10	11.1	2.8	2.8	0.46^D(b)^	0.52^DE(b)^	0.80^EF(a)^	0.72^DE(a)^	0.06^B(c)^	0.47^D(bc)^	0.63^CDE(b)^	0.87^D(a)^	0.41^D(c)^	0.09^C(d)^
Mean				0.73	0.74	1.26	0.91	0.13	0.83	0.88	1.69	0.93	0.33

‡‡ Each value is the mean of five replicates. ‡ *Ganoderma* isolates tested: *G. australe* G30 (Ga), two *G. boninense* isolates – G14 (Gbp) and G8 (Gbf), one *Ganoderma* species (G31), as well as *G. lucidum* G32 (Gl). *Values in each column represent the mean radial mycelial growth rate (mm/day). Each *Ganoderma* species or isolate was analysed separately. Means within each column for the respective runs, combinations of sugar compounds, or treatments followed by the same letter (upper case letter) (superscript) are not significantly different at *P *≤ 0.05 after ANOVA–Tukey tests were performed. †Values in each horizontal row represent the mean radial mycelial growth rate (mm/day) for the respective runs or combinations of sugar compounds. Each run, combinations of sugar compounds, or treatments were analysed separately. Means within each horizontal row followed by the same letter – lower case letter, superscript, and in the bracket () – are not significantly different at *P *≤ 0.05 after ANOVA–Tukey tests were performed.

### Statistical analyses

An augmented simplex centroid design for three sugar components (comprising glucose, sucrose, and fructose) with an initial concentration of 3.3 g/100 mL (3% w/v) for low total sugar (Table 1) and 16.7 g/100 mL (16.7% w/v) for high total sugar (Table 2) was used to establish all the sugar compositions required for each of the respective runs. Data sets obtained from the mixture design were analysed and contour plots were generated using Minitab 16. Means of radial mycelial growth rate (mm/day) for those treatments that were not normally distributed were log_10_-transformed prior to analysis of variance (ANOVA) following the approach used by Liu and Chen (2002). Media with low and high sugar concentrations, as well as between pH 5 and 8 were analysed separately. Means were then separated using Tukey’s simultaneous test at *P *= 0.05. Numbers presented in tables are untransformed means. In addition, differences in means for radial mycelial growth rate of all the tested *Ganoderma* isolates were analysed using ANOVA-GLM (general linear model (GLM)) method (Minitab 16) to assess the interactions between treatments. Fungal growth for all the *Ganoderma* isolates on media with only glucose, sucrose, or fructose was ranked based on the radial mycelial growth rates on the respective sugar components. The data were then combined and subjected to cluster analysis (Minitab 16).

### Phylogenetic analysis

Sequences of internal transcribed spacer (ITS) region for all the tested *Ganoderma* isolates were retrieved from GenBank and aligned using Clustal W (Thompson et al. 1994). Alignments are available on treebase.org at the following link http://purl.org/phylo/treebase/phylows/study/TB2:S17450. Neighbour joining (NJ) analysis was conducted using MEGA6 software (Tamura et al. 2013). The robustness of the trees was confirmed using bootstrap analyses with 1000 repetitions. Phylogenetic trees were prepared with sequences showing bootstrap values higher than 50%. Trees based on ITS sequences were rooted with sequences from *Tomophagus colossus* (Fr.) Murrill isolates (KJ143923 and JN184396).

## Results and discussion

### Effect of sugar source on radial mycelial growth

Low and high concentrations of sugars were selected based on the total sugars recorded and analysed previously in oil palm and coconut by various researches. Chooklin et al. (2011) reported the total sugars for oil palm sap were 19.17 g/L or 1.92% (w/v) and comprised only glucose (1.66%) and fructose (0.26%). In a separate study, saps from oil palm trunk were reported to have 11% total sugars (Eze and Ogan 1988). Goh et al. (2013) reported sawdust extracted from oil palm and coconut trunks contained approximately 2.77% and 21.2% w/w total sugars, respectively. Therefore, total sugar concentration of 3.3% (w/v) or 33 g/L and 16.7% (w/v) or 167 g/L (comprising only one, two, or all three major sugars – glucose, sucrose, and fructose) were used to prepare media with different combinations of low as well as high total sugar concentrations, respectively (Tables 1 and 2).

A preliminary analysis of the data on radial mycelial growth of all the *Ganoderma* isolates showed that *G. lingzhi* G32 grew the slowest in most of the runs at pH 5 and 8 compared to other *Ganoderma* isolates studied (Tables 1 and 2). In previous studies, *G. boninense* G14 (Gbp) was found to grow faster compared to *G. boninense* G8 (Gbf) and a few other *Ganoderma* isolates on MEA and other media (Goh et al. 2013; Kok et al. 2013). In the current study, *G. boninense* G14 remained the fastest grower compared to the other four *Ganoderma* isolates (Tables 1 and 2). *Ganoderma* growth rates were significantly higher (*P < *0.05, *t*-test) when inoculated on media with low sugar concentrations compared to media with high sugar concentrations at pH 5 and 8 for most of the isolates tested, except for *G. australe* G30 at pH 5, which was statistically insignificant (*P *= 0.74, *t*-test).

Results obtained from low and high sugar concentrations were analysed separately using GLM. All three factors (pH: 5 and 8; Isolate: Ga, Gbc, Gbp, Gbf, and Gl; and Sugar combinations) and the interactions between pH x Isolate, pH x sugar combinations, isolate x sugar combinations, and pH x isolate x sugar combinations had significant effects on the fungal radial mycelial growth rate (*P *< 0.05, ANOVA-GLM).

**Figure 1.**
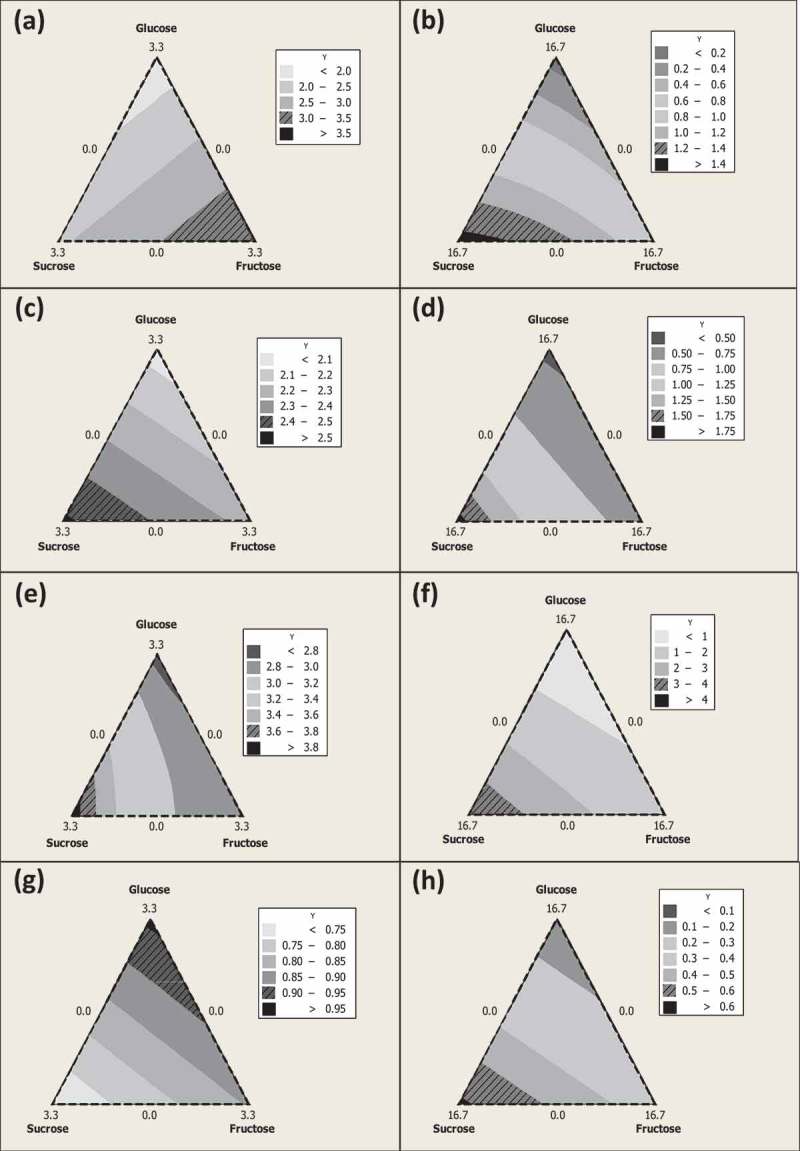
Contour plots of radial mycelial growth (mm/day) for *G. australe* G30 (a), (b); *Ganoderma* sp. G30 (c), (d); *G. boninense* G14 (e), (f); and *G. lingzhi* G32 (g), (h) inoculated on media with low (a, c, e, and g) and high (b, d, f, and h) sugar concentrations adjusted to pH 8.

Under high sugar concentrations, all tested *Ganoderma* isolates grew faster on media with sucrose, followed by fructose and glucose at pH 5 and 8 (Table 2, Figure 1 only show results for media adjusted to pH 8). Similar results were obtained when *G. boninense* G14 and 8 isolates were inoculated on low sugar concentrations at pH 5 and 8. However, at low sugar concentration, *Ganoderma australe* G30 proliferated better on media added with fructose, followed by sucrose and then glucose, while *G. lingzhi* G32 had a higher growth rate on media with glucose, followed by fructose and then sucrose. In a study by Jo and associates (2009), growth of *G. applanatum* on media with low glucose levels (1:1–5:1, glucose to sodium nitrate) was better compared to media with high glucose levels (10:1–50:1, glucose to sodium nitrate). Media amended with high glucose concentrations were inhibitory to *Ganoderma* mycelial growth. Other sugar sources, in particular, sorbitol, mannitol, fructose, sucrose, dextrin, and mannose (in descending order in terms of growth rate), were reported to result in better growth for *G. applanatum* compared to glucose (Jo et al. 2009). In a separate study, dextrin was the most preferred source for *G. lucidum* growth, followed by galactose and fructose, but glucose also resulted in the slowest growth (Jayasinghe et al. 2008). Information on the responses of five *Ganoderma* isolates to three different simple sugars was used for cluster analysis.

Based on the results generated using the mixture design, all three components (A: Glucose, B: Sucrose, and C: Fructose) posed significant positive effect on radial mycelial growth in all the five *Ganoderma* species and isolates (*P* < 0.05) for each of the individual components on media with both low and high sugar concentrations. Under media with high sugar concentrations, the response of fungal mycelial growth towards three different simple sugars for all the tested *Ganoderma* species and isolates appeared to be in similar order, with sucrose to be the highest, followed by fructose, and then glucose (B > C > A) (equations not shown here). However, the order of responses towards three simple sugars under low sugar concentration varied between tested *Ganoderma* species and isolates, for instance, *G. australe* appeared to grow better on fructose (C), *G. boninense* was found to be faster on sucrose (B), and *G. lingzhi* was observed to be better on glucose (A) (Equations (1)–(10)). The equations with second-order terms were verified and selected based on Akaike Information Criterion (AIC). The models of (1), (8), and (9) were kept based on AIC analysis.

Antagonistic and synergistic influences between simple sugars used on the *Ganoderma* growth rates were observed in Equations (1), (8), and (9). Suppression in fungal growth rate in the media with combinations of two components, especially with reducing sugars – glucose and fructose, was observed. Non-reducing sugars (e.g. sucrose) were proposed not to produce antimicrobial or inhibitory compounds when autoclaved in the presence of phosphate or other elements, whereas the reducing sugars were reported to generate inhibitory or bacticidal compounds when autoclaved with phosphate or other components (Finkelstein and Lankford 1957; Byrd et al. 1999). In a separate study, autoclaved sucrose was found to cause less inhibitory effects on the growth of carrot root tissue cultures compared to both autoclaved glucose and fructose (Stehsel and Caplin 1969). The toxic materials were not present or produced if the reducing sugar solution was filter-sterilized (Baumgartner 1938). Furthermore, bactericidal effects due to autoclaved glucose in the buffer adjusted to alkaline pH appeared to be more detrimental compared to autoclaved glucose in the buffer adjusted to pH 7 (Yoneyama et al. 2007). In the same study by Yoneyama et al. (2007), the growth and survival rates of different bacterial species tested were varied when treated with autoclaved glucose. Certain bacterial strains appeared to be more sensitive towards autoclaved glucose compared to others. More studies have to be conducted to evaluate the variations in the level of tolerance by different *Ganoderma* species or isolates towards inhibitory effect due to the toxic materials released by autoclaved sugars as well as to assess the difference in the toxic compounds produced adjusted to both acidic and alkaline pH.

Equations:

*G. australe* G30

(1) Low sugar,pH5: Growth rate= (0.65)A + (0.79)B+(1.61)C−(1.63)AC−(2.22)BC

(2) Low sugar,pH8: Growth rate= (1.71)A+(2.42)B+(3.51)C

*Ganoderma* sp. G31

(3) Low sugar,pH5: Growth rate= (1.68)A+(1.76)B+(1.57)C

(4) Low sugar,pH8: Growth rate= (2.05)A + (2.52)B+(2.27)C

*G. boninense* G14

(5) Low sugar,pH5: Growth rate= (2.47)A + (2.86)B+(2.59)C

(6) Low sugar,pH8: Growth rate=  (2.75)A + (3.78)B+(2.81)C

*G. boninense* G8

(7) Low sugar,pH5: Growth rate= (1.41)A + (1.71)B+(1.62)C

(8) Low sugar,pH8: Growth rate= (2.16)A + (3.53)B+(2.59)C−(3.64)BC

*G. lingzhi* G32

(9) $$\begin{array}{l} \text{Low}\;\text{sugar},\text{pH5}:\;\text{Growth}\;\text{rate}=\;\left(\text{0}.\text{94}\right)\text{A}+\left(\text{0}.\text{61}\right)\text{B}\\ \;\;\;\;\;\;\;\;\;\;\;\;\;\;\;\;\;\;\;\;\;\;\;\;\;\;\;\;\;\;\;\;\;\;\;\;\;\;\;\;\;\;\;\;\;\;\;\;\;\;\;\;\;\;\;\;\;\;\;\;\;\;\;\;\;\;\;+\left(\text{0}.\text{67}\right)\text{C}+\left(\text{1}.\text{16}\right)\text{AB} \end{array}$$

(10) $$\begin{array}{l} \text{Low}\;\text{sugar},\text{pH8}:\;\text{Growth}\;\text{rate}=\;\left(\text{0}.\text{96A}\right)+\left(\text{0}.\text{70}\right)\text{B}\\ \;\;\;\;\;\;\;\;\;\;\;\;\;\;\;\;\;\;\;\;\;\;\;\;\;\;\;\;\;\;\;\;\;\;\;\;\;\;\;\;\;\;\;\;\;\;\;\;\;\;+\left(\text{0}.\text{85}\right)\text{C} \end{array}$$

### Cluster and phylogenetic analyses

**Figure 2.**
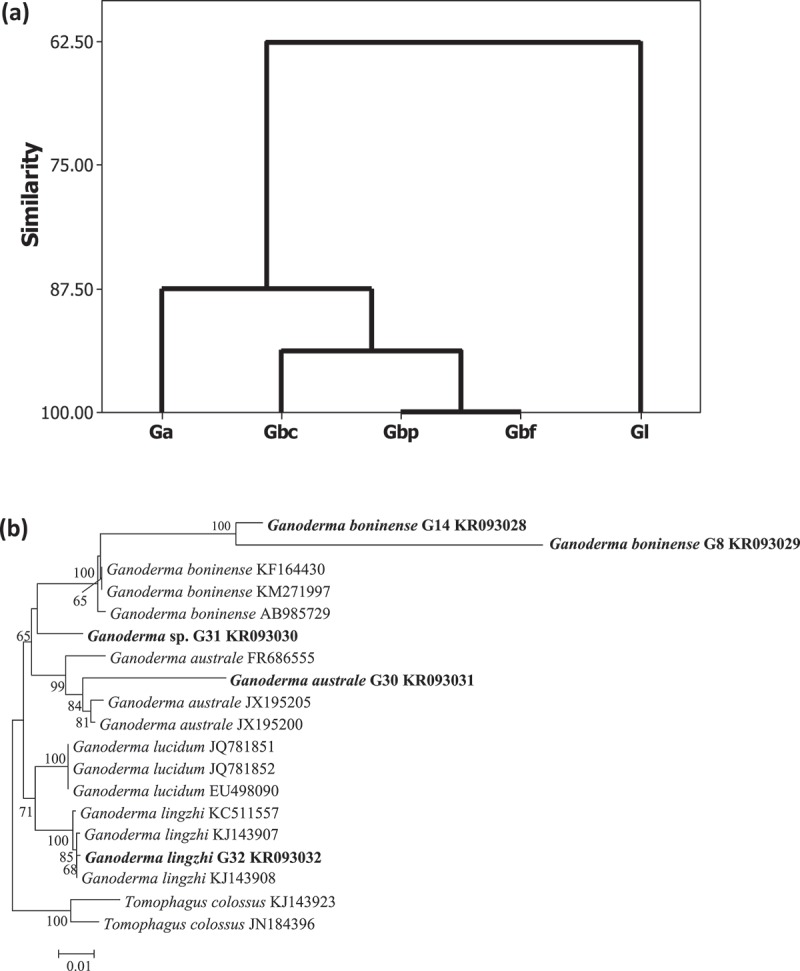
Cluster analysis based on response of five *Ganoderma* isolates to three different simple sugars (a) and phylogenetic analysis with the internal transcribed spacer (ITS) sequences for five tested *Ganoderma* isolates (*G. boninense* G14 KR093028, *G. boninense* G8 KR093029, *Ganoderma* sp. G31 KR093030, *G. australe* G30 KR093031, and *G. lingzhi* G32 KR093032) as well as 12 other *Ganoderma* sequences retrieved from GenBank (b).

Production of polysaccharides and intracellular sugar composition was proposed to be an additional parameter for determining the strains of *G. lucidum* (Stajić et al. 2011). Variations in mycotoxins produced by different *Fusarium graminearum* chemotypes on media with different sucrose concentrations were used as chemotaxonomic tool for categorizing chemotypes of *F. graminearum* (Vujanovic and Ben Mansour 2011). In the current study, data sets from the responses of five *Ganoderma* isolates in relation to mycelial growth rate on media with the respective simple sugars, low and high sugar concentrations as well as pH 5 and 8, were used for cluster analysis (Figure 2(a)). The cluster trees showed the similarity or relatedness between different *Ganoderma* species or isolates. Both *G. boninense* isolated from oil palms (G14 and 8 or Gbp and Gbf) were grouped together, and *Ganoderma* sp. isolated from coconut (G31 or Gbc) had higher similarity to G14 and 8 compared to *G. australe* and *G. lingzhi* (Figure 2(a)). A phylogenetic tree was generated with NJ (analysis using sequences of the five *Ganoderma* isolates or species used in this study, 12 other *Ganoderma* sequences (*G. boninense, G. australe, G. lucidum*, and *G. lingzhi*) and sequences of two *Tomophagus colossus* isolates retrieved from Genbank and the tree also demonstrated the same grouping with *G. boninense* from oil palms (G8 and G14 isolates) and *Ganoderma* sp. G31 from coconut in close proximity (Figure 2(b)), while both *G. australe* (G30) and *G. lingzhi* (G32) were either a distance away or branched from *G. boninense* (Figure 2(a) and (b)). *Ganoderma lucidum* and *G. lingzhi* sequences were used in this analysis to determine the identity of G32 isolate. Based on the papers by Cao et al. (2012), Yang and Feng (2013), and Dai et al. (2013), *G. lingzhi* was mainly reported in East Asia (warm temperate and subtropical regions) with pale yellowish to sulphur yellowish pore surface, whereas *G. lucidum* was commonly isolated from Eurasia (Europe) continent with a cream to whitish pore surface. In the same studies, both *G. lingzhi* and *G. lucidum* were observed to form separate clades (Cao et al. 2012; Yang and Feng 2013). Similar to the observation recorded in the current study, *G. lingzhi* G32 and a few other *G. lingzhi* isolates formed a separate clade and branched from *G. lucidum* isolates (Figure 2(b)). More *Ganoderma* species and isolates would have to be included for future studies to determine whether mycelial growth rate on different sugar media can be incorporated as one of the additional criteria for separating different *Ganoderma* species.

## Conclusions

Media with high total sugar concentrations were inhibitory to all tested *Ganoderma* species. In addition, under high total sugar condition, all tested *Ganoderma* species grew the fastest on media with sucrose, followed by fructose and glucose. In the media with low total sugar concentrations, variation in sugar preferences was observed with *G. australe* (pathogen of *Schizolobium parahybum*) and *G. lingzhi* (medicinal mushroom) preferring fructose and glucose, respectively. On the contrary, *G. boninense* isolates (oil palm BSR pathogens) and *Ganoderma* species (from coconut palm) showed high affinity towards sucrose. With equations generated using the mixture design approach, antagonistic and synergistic effects between the studied sugars on *Ganoderma* growth were illustrated. Based on cluster and phylogenetic (ITS sequences) analyses, *G. boninense* G14 and G8 were located within the same clade as other *G. boninense* isolates, followed by *Ganoderma* species, and then *G. australe* clade, whereas the most distant clade was *G. lingzhi*. However, more *Ganoderma* species and isolates shall be incorporated to assess the use of mycelial growth rate on media with different sugar combinations for separating *G. boninense* from other *Ganoderma* species.

## Disclosure statement

No potential conflict of interest was reported by the authors.
